# Calcific Uremic Arteriolopathy on Multimodal Combination Therapy: Still Unmet Goal

**DOI:** 10.1155/2012/390768

**Published:** 2012-02-20

**Authors:** Usman Hammawa Malabu, Valli Manickam, George Kan, Susan Lynette Doherty, Kunwarjit Singh Sangla

**Affiliations:** ^1^Department of Endocrinology, The Townsville Hospital, 100 Angus Smith Drive, Douglas, QLD 4814, Australia; ^2^Renal Medicine, The Townsville Hospital, 100 Angus Smith Drive, Douglas, QLD 4814, Australia; ^3^Occupational Therapy, The Townsville Hospital, 100 Angus Smith Drive, Douglas, QLD 4814, Australia

## Abstract

*Background*. Calcific uremic arteriolopathy (CUA) or calciphylaxis though generally noted for its high mortality, recent case reports have shown promising results using single agent therapies. However, it is not clear whether combination therapeutic agents will improve course of the disease. *Objective*. To determine clinical outcome in subjects with CUA on multimodal treatment. *Methods*. All patients with end-stage renal failure (ESRF) at The Townsville Hospital, Australia, from April 1, 2006, to March 31, 2011, with diagnosis of CUA were retrospectively studied. *Results*. Six subjects with CUA (4 females and 2 males) were on various combination therapeutic agents comprising sodium thiosulphate, hyperbaric oxygen, prednisolone, cinacalcet, and parathyroidectomy in addition to intensified haemodialysis, specialist local wound care, and antibiotics. The wounds failed to heal in 3 patients while 5 of the 6 subjects died; cause of death being sepsis in 3 and myocardial infarction in 2. *Conclusion*. Prognosis of CUA remains poor in spite of multimodal combination therapy. Further prospective studies on a larger population are needed to verify our findings.

## 1. Introduction

Calcific uremic arteriolopathy (CUA) or calciphylaxis is a syndrome of painful skin necrosis and vascular calcification with high morbidity and mortality. It occurs primarily in patients with end-stage renal disease [1, 2]. Though generally a rare syndrome, it is increasingly described in subjects undergoing renal dialysis. Calciphylaxis has also been described in subjects with normal renal function including primary and secondary hyperparathyroidism [3, 4]. A common factor linking non-renal-failure-related presentation of the same disease is elevated parathyroid hormone with or without elevated calcium-phosphate product leading to calcification of small vessels. The consequences of these are significant mortality of 80% principally from multiple end organ damage due to ischemia and infarction commonly complicated by infection; sepsis being the principal cause of death [1–3]. Thus, traditional care addresses the calcium-phosphate-PTH axis: substituting calcium with non-calcium as phosphate binders, strict dietary phosphate control, cautious vitamin D analogs, calcimimetics, and surgical parathyroidectomy if necessary. Newer approaches focus on intravascular and tissue mineralization: dissolution of calcium deposits with the use of sodium thiosulfate, altering osteoprotegerin, and NF-*κ*B pathways with bisphosphonates [4, 5]. Recently, single agent treatment such as sodium thiosulphate and hyperbaric oxygen has yielded various successes in care of calciphylaxis [5–7]. Furthermore, favourable outcome was recorded in limited reports on CUA using cinacalcet or parathyroidectomy to achieve targeted normalised calcium, phosphate, and parathyroid hormone levels [8, 9]. Yet usefulness of these agents in the form of combination therapy has not been fully explored [10, 11]. The aim of the study was to review all cases of CUA seen in the last five years and determine its clinical course in the setting of multimodality care.

## 2. Material and Methods

All patients diagnosed to have CUA at The Townsville Hospital, Australia, from April 1, 2006, to March 31, 2011, were retrospectively studied. Subjects with nonuremic calciphylaxis were excluded from the study. Approval for the study was sought from the hospital's ethical committee. Data collected included age, gender, onset of dialysis to diagnosis of calciphylaxis, duration of the skin lesion, and outcome defined as completely or partially healed skin lesions or death of a subject. In all subjects, calciphylaxis trigger agent(s) were ceased—calcium-based phosphate binders and alphacalcidol; none was on warfarin. All the patients were on oral erythropoietin for anemia in addition to iron infusion in 2 patients. Primary cause of the renal failure was also determined as well as comorbidities. Biochemical profile of subjects with CUA at diagnosis and within 6 months on multimodal care was recorded. These included corrected calcium, phosphate, albumin, calculated calcium-phosphate product, and parathyroid hormone.

The patients included in this study were reviewed by the vascular team, and a second opinion was obtained from a dermatologist. All lesions had a punch biopsy performed across the ulcer edge, but if the specimen was insufficient to demonstrate calciphylaxis, then a wedged biopsy was performed to confirm the diagnosis. All biopsies were analysed by the same pathologist; the diagnostic criteria used were medial calcification of dermal small and medium-sized arterioles and arteries, intimal fibroblastic and endothelial proliferation associated with ischemic necrosis of subcutaneous adipose tissue, and epidermal ulceration. Medium-sized arteries with medial calcific sclerosis (Monckeberg's medial calcific sclerosis) with no intimal or endothelial proliferation were excluded from the study.

The patients' wounds were managed by clinical occupational and nurse therapist with specialist interest in wound healing and compression therapy. The dry necrotic lesions were gently hydrated to promote a moist wound environment, encouraging autolytic debridement and cell migration. An atraumatic dressing (silicone coated, Mepitel) and nonadherent absorbent pad were applied as secondary dressings. After autolytic debridement, a conformable silver polymembrane was applied and retained with light tubular support bandage. If the wound was infected, antibiotics were administered based on microscopy, culture, and sensitivity results following deep wound swab and blood cultures with input from the infectious disease specialist. To improve wound healing, daily hyperbaric oxygen was used in some patients. Each treatment lasted 90 minutes undertaken at 2.4 atmospheres absolute (ATA).

Methods used in lowering calcium-phosphate product and parathyroid hormone included emergency parathyroidectomy, use of non-calcium phosphate binders—sevelamer 4.5 g/day—and cinacalcet 30 mg daily. The latter being calcimimetic lowers parathyroid hormone by increasing sensitivity of the calcium receptor. All the patients had low calcium containing dialysate (calcium concentration 1.25 mEq/L) and the dialysis-dose was increased from 8 L dialysate/day to 12 L/day. Oral prednisolone 50 mg once daily was administered in 2 patients due to contraindication to parathyroidectomy and refusal to use sodium thiosulphate (STS). Twenty-five grams of STS (100 mL of a solution at 25% STS) were infused three times per week in 3 patients for 1 to 3 months. The thiosulphate was administered immediately after dialysis. There were no signs, symptoms, or electrocardiographic evidence of hypocalcaemia at any time during the course of STS therapy. There was no discernible effect of each thiosulphate infusion on any of the plasma chemistry values or any other adverse effects such as metabolic acidosis or haemorrhagic complications. Wound response to the treatments was monitored clinically and by serial photography comparing before and after progress. In addition, patients were followed up in outpatient clinics to determine if the healed skin lesion relapsed or to determine cause of death had the patient died. Treatment for comorbidities was provided and followed up by the appropriate subspecialties.

Once discharged from the hospital, patients were followed up weekly by the clinical occupational therapist and by nephrologists 3 times a week at the renal dialysis session looking for relapse of the calciphylaxis. Results were presented in tabular form for all the patients. Comparison of biochemical profile was made at and within 6 months after the diagnosis of CUA with overall results depicted as mean ± standard deviation, where applicable.

## 3. Results

Over the five-year period, we recorded 6 cases of CUA out of 201 subjects who had regular haemodialysis sessions at the unit as shown in Table 1. Of the 6 subjects, two-third was females. Mean duration of dialysis was 20 months range 1 to 3 years. Infection was demonstrable in all subjects as revealed by wound swab microscopy, culture, and sensitivity. The commonest organism isolated was *Pseudomonas aeruginosa* in 4 subjects, namely, patient 1, 4, 5, and 6. Patient 6 in addition had methicillin resistant *Staphylococcus aureus* (MRSA). Patient 2 and 3 had MRSA and *Klebsiella pneumonia,* respectively. Modalities of treatment of the calciphylaxis apart from wound care and administration of antibiotics in all included cinacalcet in patient 1, 3, and 6; prednisolone was used in patient 3 and 4, while parathyroidectomy was emergently done in patient 2 and 3 with marked hyperparathyroidism and progressive disease despite medical therapy. Following the parathyroid surgery, whereas patient 2 had her wounds completely healed and remained in remission for 32 months, patient 3 did not respond to the parathyroidectomy and died 5 months later of sepsis. Below knee amputation was performed in only one subject, patient 5, who did not respond to local wound care, antibiotics, hyperbaric oxygen therapy, and administration of sodium thiosulphate. The CUA reoccurred rapidly within one week of the surgery which was complicated by sepsis following withdrawal of further treatment including haemodialysis. He died one month after the knee amputation. Side effects of the treatments offered were assessed, but none of the patients developed any untoward effects to prednisolone, cinacalcet, STS, or hyperbaric oxygen therapy. Overall, combination therapy for CUA was offered in the form of dual in 4 subjects, triple and quadruple in one subject each as detailed in Table 3.

In addition to ESRF, other diseases were recorded premorbidly. These include ischemic heart disease in subject 1 and 4 though patient 2 and 4 both died suddenly, and autopsy confirmed myocardial infarction as a cause of death. Other comorbidities apart from diabetes in all but 2 patients, hypertension occurred in subject 1 and 5. Interestingly, patient 2 and 3 were on treatment for gout. Closer look at the survival details revealed only one patient is alive, the rest died mainly within 9 months of diagnosis of CUA.

**Figure 1:**
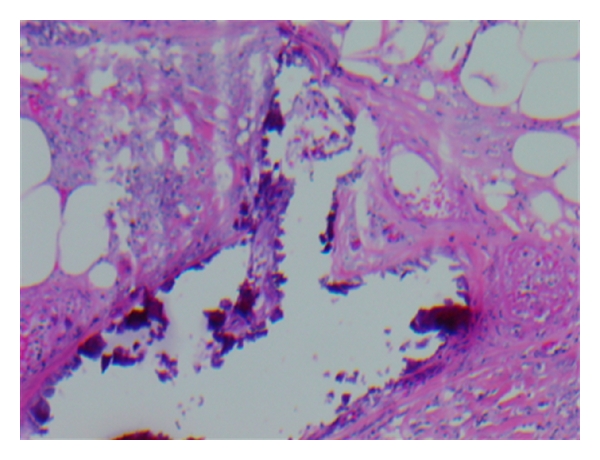
Histology of skin biopsy of patient 4 showing calcific arteriolopathy typical of calciphylaxis. Haematoxylin and eosin stain, magnification 200x.

The histology of the skin biopsy of the patients was analysed histologically as depicted in Figure 1. The defining histological characteristic found in all the cases was extensive calcification of small and medium-sized vessels of the dermis and subcutis layer with typical calcific thrombogenic microangiopathy. The deposition of calcium in the media was either segmental or circumferential and was accompanied by intimal hyperplasia, fibrosis, and thickening with smooth muscle fibre atrophy.  Table 2 shows mean biochemical profile of subjects with CUA at diagnosis and within 6 months after. Calcium-phosphate product was similar in both groups, while serum parathyroid hormone was significantly lower after the diagnosis.

**Figure 2:**
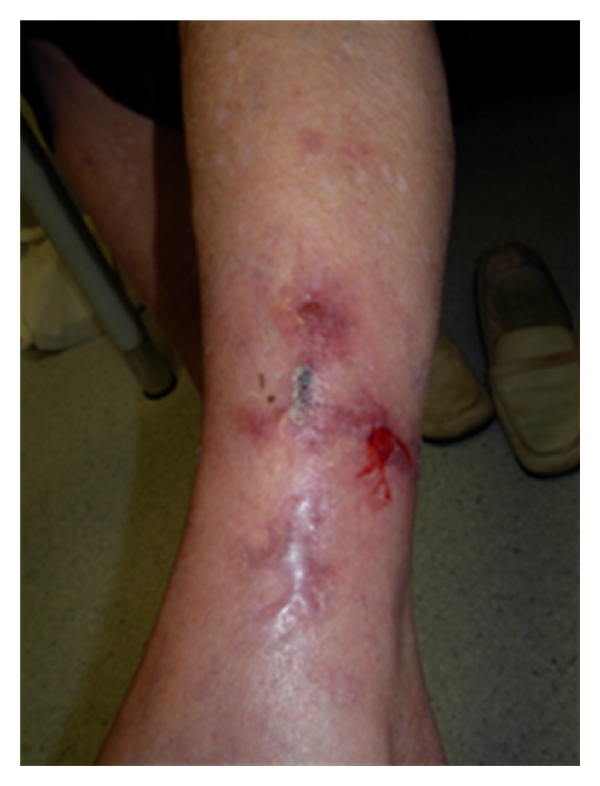
Successfully healed calciphylaxis following autolytic debridement and dressings along with treatment with cinacalcet and prednisolone in patient 4 who later died of myocardial infarction.

Figure 2 shows completely healed ulcers of calciphylaxis in patient 4 who had been on treatment for ischemic heart disease. She presented initially with extremely painful leg ulcers with features of cardiac failure. Further echocardiographic assessment revealed extensive coronary calcification involving 3 main vessels. She was declared not for coronary bypass grafting and opted for palliative care. She was on the following oral agents: aspirin 100 mg, clopidogrel 75 mg, nicorandil 20 mg, isosorbide mononitrate 120 mg, frusemide 40 mg, atenolol 50 mg, candesartan-hydrochlorothiazide 16/12.5 mg, atorvastatin 40 mg, and amlodipine 10 mg all were given once daily. Daily wound care and oral cinacalcet along with prednisolone were commenced to which she responded dramatically within one month and the lesions completely healed by second month as shown in Figure 2. She remained symptomatic of cardiac angina despite on multiple medications. Sadly, she died suddenly and autopsy confirmed myocardial infarction as the cause of death. On the other hand, patient 1 had painful bilateral legs lesions which progressed rapidly within a month with involvement of genitalia and breast. Sodium thiosulphate infusion and cinacalcet were given for one month without improvement. The patient declined further care and died of sepsis a week later following discontinuance of active treatment including haemodialysis.

All the patients had ESRF on haemodialysis; two-third due to diabetes. Other causes of ESRF recorded include chronic glomerulonephritis and obstructive uropathy in one subject each, respectively. All the subjects had lower limb skin lesions. In addition, both patients 1 and 5 had proximal involvement. The skin lesion partially healed in one subject and failed to heal in the 3 who died of overwhelming sepsis. On the other hand, the 2 subjects with successfully healed wounds died of myocardial infarction 1 to 2 years later.

## 4. Discussion

We have demonstrated poor outcome of calciphylaxis in subjects on multimodal combination therapies. All except one patient died within an average of 9 months from the diagnosis of the skin lesion; in line with others observation [1, 2]. Recently, reports have shown high survival rate and complete wound healing with the use of single agent therapy including sodium thiosulphate, hyperbaric oxygen, cinacalcet, and parathyroidectomy [5–9]. Surprisingly, such benefit was not evident in our subjects on multiagent treatment in the form of dual, triple, or quadruple therapies, similar to Kyritsis et al. observation [10], but in contrast to better outcome noted by Arenas et al. and by Baldwin and colleagues [11, 12].

It is interesting to note that though single or multiple-agent therapies aimed at lowering calcium-phosphate product have shown some positive results in treatment of calciphylaxis, to-date no standard management is universally accepted to treat this condition. Nonetheless, non-calcium-containing phosphate binders, bisphosphonates, the calcimimetic cinacalcet, and vitamin D analogs have been used with this intent [13]. For instance, pamidronate, a bisphosphonate used singly or in combination with other agents, has been reported to respond favourably in subjects with calciphylaxis [4, 14]. Similarly, by lowering serum parathyroid hormone, cinacalcet has also proved to be beneficial in care of CUA [15]. Furthermore, emergency parathyroidectomy has been used in the treatment of patients with CUA associated with hyperparathyroidism refractory to medical therapy; however, case series have shown variable results [1, 9, 16]. Other treatment modalities include hyperbaric oxygen therapy which has been found useful in management of CUA by improving tissue oxygenation and promoting wound healing [7, 17]. Similarly, sodium thiosulfate increases solubility of calcium deposits in CUA with enhanced wound healing [6]. The success of sodium thiosulfate therapy alone or in combination with other agents in patients with calciphylaxis has been described in several case reports and case series [5, 18, 19]. Despite the observation by others of favourable outcome using multiple agents in care of CUA, we recorded high mortality even after successful wound healing.

The factors attributable to the poor prognosis of CUA in our series are not clear. Possible reasons for the high mortality include delayed diagnosis due to low index of suspicion emanating from alternative diagnosis, including more common diabetic skin ulcer and osteomyelitis; both conditions may coexist with the calciphylaxis itself [20]. Furthermore, other skin lesions such as vasculitis may mimic calciphylaxis clinically including painful nonhealing ulcers due to ischemia which might have led to delayed diagnosis. However, with the high index of suspicion of CUA in our cohort makes it less likely as a contributing factor. On the other hand, site of CUA lesion has been reported to have prognostic value. Some authors believe proximal localization of the lesion to have worse prognosis [1, 21]. Interestingly, only 2 of our subjects had proximal involvement yet 83% of them died despite multi-interventional approach; in agreement with others findings [1, 2]. Another confounding prognostic factor is comorbidities. Five of the 6 subjects studied had at least 3 other diseases apart from the CUA to cope with. Furthermore, the cause of the calciphylaxis may have prognostic significance. Nonuremic calciphylaxis tends to have a less ominous course compared to those with ESRF [3, 4] in support of high mortality in our study in which all the subjects had renal failure from various aetiologies on haemodialysis.

The major limitation of our study was small sample size which limits power to detect associations reflecting relative rarity of this condition in clinical practice. Furthermore, the biochemistry profile presented was limited to at the time of diagnosis and within 6 months after and did not address daily changes which may have occurred in the course of followup of the subjects. Nonuniformity of treatment of calciphylaxis also makes it hard to conclude outcome as poor as reported. Furthermore, contribution of other risk factors of ischemic heart disease was not assessed in spite of myocardial infarction being one of the common causes of death in the study population. Lastly, it is important to note that there is a limitation to retrospective studies in general. Observations derived from such studies may contain some missing information and thus may serve as a stimulus to further prospective work to clarify findings. The present work must be interpreted in the knowledge of the defects inherent in such studies. Despite these, our result is consistent with other reports [2, 10]. The study has further suggested calciphylaxis, a syndrome with high mortality irrespective of mode of care.

In conclusion, prognosis of CUA in subjects with ESRF remains poor in spite of combination therapy and multimodality of wound care. Further prospective studies on a larger population are needed to characterise our findings.

## Figures and Tables

**Table 1:** Patients' characteristics and clinical course in subjects with end-stage renal failure presenting with calciphylaxis.

Subject	Patient 1	Patient 2	Patient 3	Patient 4	Patient 5	Patient 6
Age (years)	48	53	63	72	74	86
Gender	Female	Female	Female	Female	Male	Male
Cause of ESRF	Diabetes	Chronic GN	Diabetes	Diabetes	Diabetes	Obstructive uropathy
Duration RRT (months)	12	36	24	21	15	12
Site of lesion	Extensive*	Distal	Distal	Extensive	Distal	Distal
Microorganism	Pseudomonas	MRSA	Klebsiella	Pseudomonas	Pseudomonas	MRSA + pseudomonas
Mode of therapy**	Cinacalcet STS	PTHX HBO	PTHX HBO STS prednisolone	Cinacalcet prednisolone	STS HBO amputation	Cinacalcet HBO
Outcome	Dead	Dead	Dead	Dead	Dead	Alive
Cause of death	Sepsis	Myocardial infarction	Sepsis	Myocardial infarction	Sepsis	Not applicable
Comorbidities	DM ESRF IHD HTN Asthma	Epilepsy ESRF gout	DM ESRF gout	IHD DM ESRF MGUS	DM ESRF HTN arthritis	ESRF
Skin lesion outcome	Failed	Healed	Failed	Healed	Failed	Healed
Survival (months)	1	32	5	4	4	Alive

ESRF: end-stage renal failure, GN: glomerulonephritis, RRT: renal replacement therapy, *: extensive skin lesions involving the lower limbs, genitalia, abdominal wall, and breast, MRSA: methicillin resistant Staph aureus, **: mode of therapy apart from general wound care and antibiotics, STS: sodium thiosulphate, PTHX: parathyroidectomy, HBO: hyperbaric oxygen, IHD: ischemic heart disease, HTN: hypertension, MGUS: monoclonal gammopathy of unknown significance.

**Table 2:** Comparison of biochemical profile of subjects with calciphylaxis at diagnosis and within 6 months after the diagnosis.

Test	At diagnosis	3 to 6 months after diagnosis	*P* value
Calcium (mmol/L)	2.3 ± 0.2	2.4 ± 0.2	NS
Phosphate (mmol/L)	2.0 ± 0.8	1.8 ± 1.0	NS
Ca × P (mmol/L)	4.5 ± 1.0	4.2 ± 1.8	NS
PTH (pmol/L)	80 ± 53	21 ± 17	<0.05
Albumin (g/L)	28 ± 7	26 ± 9	NS

SD: standard deviation, Ca × P: calcium-phosphate product, PTH: parathyroid hormone, NS: not significant.

**Table 3:** Multimodal care and outcome in subjects with calcific uremic arteriolopathy.

Patient	Antibiotics	Sodium Thiosulfate	Cinacalcet	Hyperbaric Oxygen	Prednisolone	Parathyroidectomy	Outcome
1	*√*	*√*	*√*				Dead
2	*√*			*√*		*√*	Dead
3	*√*	*√*		*√*	*√*	*√*	Dead
4	*√*		*√*		*√*		Dead
5	*√*	*√*		*√*			Dead
6	*√*		*√*	*√*			Alive
